# Assessment of orthodontic borderline treatment need: A comparison of two aesthetic indices

**DOI:** 10.1186/s40510-022-00419-2

**Published:** 2022-07-25

**Authors:** Ariane Sampson, Neha Passan, Huw G. Jeremiah, Robert Kirschen

**Affiliations:** 1 Department of Orthodontics, Clinic 8, Addenbrooke’s Hospital, Cambridge University Hospital NHS Foundation Trust, Hills Road, Cambridge, CB2 0QQ UK; 2 Northlight Dental, 6 The Square, Aspley Guise, Bedfordshire, MK17 8DF UK; 3 Department of Orthodontics, Institute of Dentistry, Barts and The London School of Medicine and Dentistry, Queen Mary University of London, Whitechapel, London, E1 2AD UK

**Keywords:** IOTN, Orthodontic treatment need, Perception, Malocclusion, Borderline, Aesthetics, Index, Oral health

## Abstract

**Objective:**

To compare a new “guide for borderline orthodontic need” (GBON) with the “aesthetic component” (AC) of the IOTN in assessing borderline cases (dental health component DHC 3), and to compare reliability and opinions of orthodontists on the use of each index.

**Materials and methods:**

Cross-sectional population descriptive study. Ninety-four qualified orthodontists assessed 30 borderline malocclusions according to the GBON and AC indices and completed a questionnaire.

**Results:**

Kappa analysis showed GBON and AC to have similar intra-examiner reliability (*K* = 0.64 and 0.60 ,respectively). Cronbach’s alpha inter-examiner reliability analysis showed GBON and AC to have similar, acceptable reliability (*α* = 0.7 and 0.9 ,respectively). There was only fair agreement between GBON and AC in terms of the number of malocclusions deemed as needing treatment (AC threshold 6). Analysis of specific occlusal traits revealed that reverse overjets were deemed as needing treatment according to AC but not anterior open bites. Both traits were assessed as needing treatment according GBON. Despite a lack of familiarity with GBON, assessors found GBON easier to use and more appropriate in assessing borderline malocclusions.

**Conclusions:**

Both GBON and AC had good and similar inter- and intra-examiner reliability. There was substantial agreement on treatment need between GBON and AC but only when the AC threshold is reduced to 4. GBON was more able to identify malocclusal traits in need of treatment than AC. GBON was found to be easier to use and considered more appropriate than AC in judging DHC 3 malocclusions.

## Introduction

The index of orthodontic treatment need (IOTN) [1] was developed in 1989. It consists of the dental health component (DHC) [2] and the aesthetic component (AC) [3] which help identify individuals who are most in need of, or would most benefit from orthodontic treatment. The AC was created as a scale of aesthetics, but is now used by clinicians to verify treatment need thresholds (“Appendix 1”).

There is wide acceptance of the merits of the DHC with the features of a borderline case (DHC 3) clearly stated. However, only three of the six features are present in the photos of the AC with the other three, anterior open bites, crossbites and reverse overjets, not present. It remains unknown whether some of the occlusal traits that can be seen in DHC 3 borderline malocclusions are identified fairly by the AC as needing treatment.

It is known that decisions about treatment for borderline malocclusions on the basis of appearance can be difficult [4, 5]. A prioritisation system was introduced for England and Wales in 2006 in which borderline cases with DHC score 3 and AC scores of 6 and above (known as score 3.6) [6] would have access to treatment within the National Health Service. However, the AC photographs 5 and 6 have not been validated specifically to determine whether or not they are appropriate thresholds for access to treatment. Some authors have suggested that the thresholds for access to treatment should be lowered so that grades 5 and even 4 are included [7–10].

A new index, the “guide for borderline orthodontic need” (GBON) [11] has been proposed as a means of differentiating the need for orthodontic treatment on aesthetic grounds in borderline cases (“Appendix 2”). The GBON consists of a chart of eight photographs in which all the malocclusions have a DHC score of 3. The photographs included in the GBON index were selected on the basis of unambiguous agreement by orthodontic, dental and lay panels of judges that these subjects did or did not need treatment on aesthetic grounds. The GBON is intended to help clinicians judge borderline DHC 3 cases, not to grade dental aesthetics.

The aims of this investigation were to compare the new GBON Index with the AC of the IOTN in terms of agreement between indices, reliability of the indices, and orthodontists’ perceptions of ease of use and appropriateness of these indices in the assessment of borderline malocclusions with DHC score 3.

## Materials and methods

### Ethical approval and consent

Ethical approval was obtained from the East London and the City Local Research Ethics Committee for the development of the GBON.

### Sample selection

Following statistical advice, the sample size calculation was based on the results of a pilot study. A minimum sample size for the study was determined to be 85 assessors, with an expected frequency outcome of measure of a minimum of 15%.

Ninety-four orthodontists were recruited simultaneously at the British Orthodontic Conference in Brighton. This annual conference is attended by a substantial proportion of UK orthodontists, and therefore provides a representative sample for the study. The only exclusion criteria were not possessing an orthodontic qualification or being retired from clinical practice for more than 1 year.

### Method

Thirty photographs were chosen from a pool of 100 photographs exhibiting various malocclusions with DHC score 3. The photographs were all anterior views of teeth in occlusion used in the original study that developed the GBON. Two sets of these 30 photographs were printed onto glossy A6 photo paper and placed in a photo album with one photo per page. One album was labelled “AC” and the other labelled “GB”.

In order to remove bias, half the assessors were given the AC album of photographs to judge first and the other half were given the GB album to judge first. After they assessed the photographs with their first index, they then assessed the photographs again but using the other index. The assessors were provided with copies of the AC of the IOTN and GBON indices for reference. The GBON index used for the study had been modified by deleting any reference to borderline malocclusions.

Assessors were therefore blind to the fact that the GBON index specifically assesses borderline malocclusions. They were also not given any information regarding the treatment need threshold score and were not made aware that the malocclusions being assessed were all specifically DHC 3 cases. The chief investigator (NP) remained present whilst the participants carried out their assessments.

The assessors were instructed to give AC scores on the basis of which photo is closest in “attractiveness” whereas the GBON scores were to be given on the basis of which photo in closest in “appearance”. They were also not made aware that selection of GBON photos A, C or E would signify that treatment is not considered necessary, or that photos B, D, F, G or H would indicate treatment need.

### Error study

An error study was incorporated by repeating every 3rd photograph at the end of the series of photographs. This increased the total number from 30 to 40. Assessors were not made aware they were also taking part in an analysis of repeatability.

### Questionnaire

The questionnaire consisted of one table for the AC scores, another table for the GBON scores, and questions on gender and whether they had previously attended an IOTN calibration course. Two further questions were asked verbally after completing the assessments. This was to reduce bias and prevent assessors from considering their opinions as they were scoring the photographs:

- Which index did you find easier to use?
- Which index did you find more appropriate for these particular malocclusions?

### Statistical analysis

Data from the main study and scores from the error study were analysed by the Statistical Package for Social Sciences computer software (SPSS Inc., Michigan Avenue, Chicago, IL, USA), using chi-square analysis, kappa analysis, and Cronbach’s *α* reliability analysis. A statistician was consulted regarding study design and statistical analysis.

## Results

### Difference between subgroups on treatment need decisions

Of the assessors, 62% were male and 38% were female, and 46% had previously attended an IOTN calibration course. The GBON and AC scores were compared according to gender, IOTN calibration, and which index was used first. The differences were assessed using the Chi-square test. There were no statistically significant differences between the subgroups at 95% level (*p* < 0.05). The Chi-square values ranged from *p* = 0.26 to *p* = 0.87.

### Inter-rater agreement

The reliability of each index was assessed using Cronbach’s *α* reliability scoring (Table 1). Both the GBON and AC indices had very acceptable *α* reliability scores, with the AC scoring higher.

**Table 1:** Chronbach’s *α* reliability scores for GBON and AC indices

	GBON index	AC index
Reliability (*α*)	0.7	0.9

### Agreement on treatment need using GBON and AC indices

The malocclusions considered to need treatment according to the GBON Index and the AC Index at treatment need thresholds 6, 5 and 4 are highlighted in grey in Table 2.

**Table 2:**
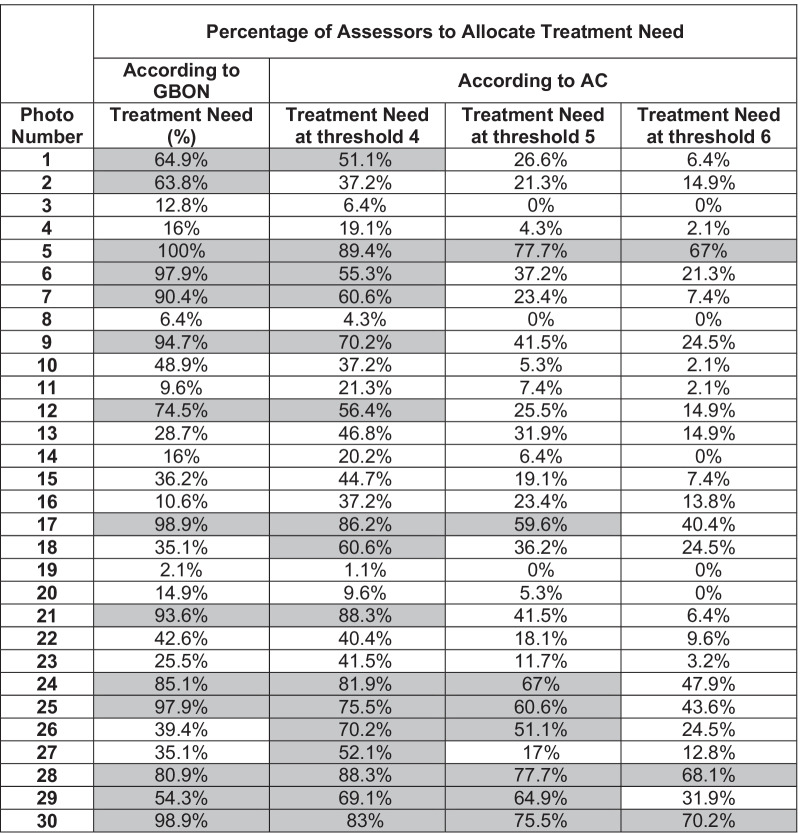
Percentage of assessors that allocated treatment need according to the GBON, compared to the AC at different treatment need thresholds

Using the GBON Index, 14 out of 30 malocclusions were considered to need treatment, whereas using the AC Index with treatment need threshold 6 (as currently used in England and Wales), just three out of the 30 malocclusions were considered to need treatment. This increased to eight malocclusions with AC threshold 5, and sixteen with AC threshold 4.

The GBON seems to have helped the assessors be decisive as, for 28 of the 30 cases, the treatment need scores were either below 40% or above 60%. They were more divided in their opinions, within the 40–60% middle ground, for only two of the 30 malocclusions. In comparison, when using the AC with threshold 4 (the most similar to GBON), eight photos were in the 40–60% range.

Agreement analysis on the percentage of malocclusions needing treatment between GBON and the three AC treatment need thresholds is shown in Table 3. The kappa agreement levels increased from fair at AC threshold 6, to moderate at threshold 5, to substantial at threshold 4.

**Table 3:** Kappa agreement score between the GBON index and the AC index at different treatment need thresholds

	GBON versus AC at threshold 4	GBON versus AC at threshold 5	GBON versus AC at threshold 6
Kappa agreement score	0.74	0.45	0.23

### Intra-rater agreement

In addition to the original 30 borderline photographs displaying DHC 3 malocclusions, 10 repeat photos were added as part of the error study. Table 4 shows that the percentage perfect agreement for the GBON and AC (threshold 6) were similar at 84% and 85%, respectively. The GBON had a kappa score of 0.64 which signified substantial intra-rater agreement. Reducing the treatment threshold of the AC index to 5 and 4 lowered the percentage agreement and kappa agreement scores. The AC kappa scores were moderate for all three thresholds.

**Table 4:** Intra-rater agreement on treatment need using the GBON index and the AC index at different treatment need thresholds

	GBON	AC at treatment threshold 4	AC at treatment threshold 5	AC at treatment threshold 6
Perfect agreement	84%	79%	80%	85%
Kappa score	0.64	0.55	0.59	0.60

### Borderline malocclusion traits not represented by the GBON or AC

Three out of the 6 borderline traits are not represented by the AC (reverse overjets, crossbites and open bites). One borderline trait is not represented by the GBON, crossbites, except as part of a reverse overjet. The results show that all of these borderline traits were considered to need treatment using the GBON or the AC at threshold 4. However, anterior open bites were not assessed as needing treatment when the AC threshold was set at 6. Table 2 shows the results for reverse overjets (photos 5 and 30), anterior open bites (photos 6 and 25) and single tooth crossbite with crowding (photo 28).

### Assessor opinions

**Fig. 1:**
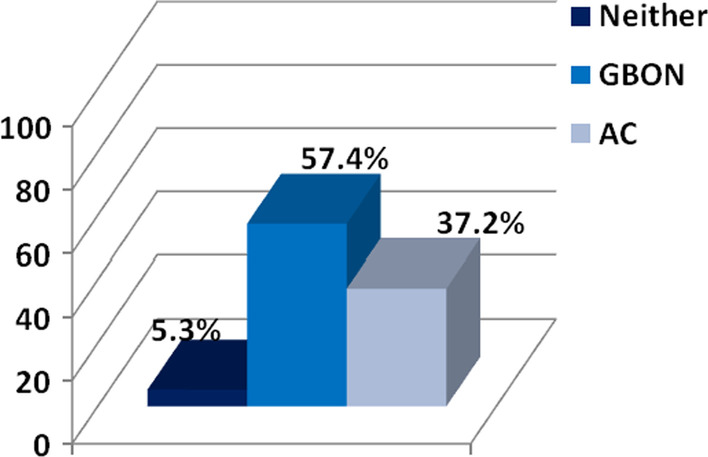
Opinions of assessors on which index was easier to use

Over half the assessors (57.4%) felt the GBON was easier to use than the AC despite never having used it before (Fig. 1). Only 37.2% preferred the AC over the GBON and 5.3% were undecided. Gender and whether assessors had used the GBON or the AC first had no influence. However, previous IOTN training had an impact as 66.7% of assessors who were not IOTN-calibrated found the GBON easier to use as compared to 46.6% for those who were calibrated (chi-square = 0.003).

**Fig. 2:**
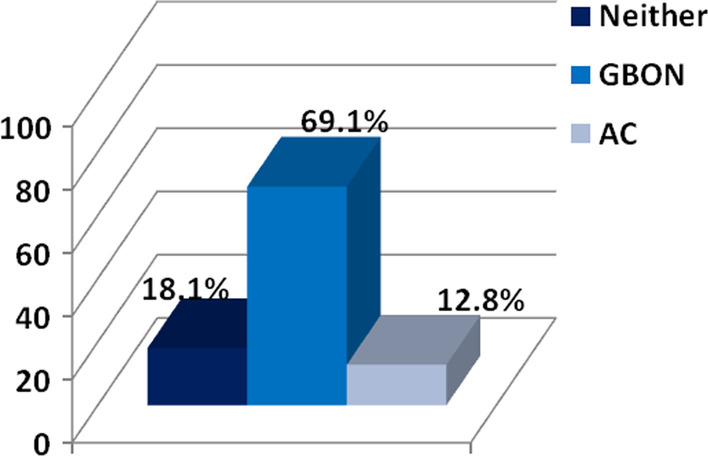
Opinions of assessors on which index was felt to be more appropriate

The majority of assessors considered the GBON to be more appropriate than the AC (69.1%), with only 12.8% considering the AC more appropriate whilst 18.1% remained undecided (Fig. 2). Gender, previous IOTN calibration, and whether assessors had used the GBON or the AC first had no influence.

## Discussion

The original purpose of the AC of the IOTN was to provide an assessment of aesthetic impairment of malocclusions from the mildest to the most severe. The AC was not created or validated for the specific purpose of discriminating which borderline malocclusions will be eligible for treatment and which ones will not. In contrast, it is appropriate to emphasise that the eight photographs selected for the GBON [11] were agreed by panels of orthodontic, dental and lay judges (75 in total) as unambiguously needing or not needing treatment. The GBON has now been shown to be an easy-to-use binary guide to indicate treatment need for borderline malocclusions based on appearance.

It is also relevant to understand that whilst the 30 borderline malocclusions assessed in this study were intended to include a range of traits, they were not necessarily representative of the prevalence of such traits in the UK child population with DHC 3 malocclusions. The intention was to test the GBON’s ability to assess all types of borderline malocclusions.

### Inter-rater agreement

To achieve validation, inter- and intra-rater agreements were examined. Both the GBON and AC indices had very acceptable Cronbach’s *α* reliability scores. It is encouraging that GBON has an acceptable *α* reliability score given that it only contains photographs within a narrow spectrum of treatment need.

### Intra-rater agreement

Intra-rater agreement was assessed by repeating every 3rd photo at the end of the series of images. The kappa test for the GBON showed substantial reliability. For the AC at treatment need threshold 6, the kappa score showed moderate reliability. When the AC treatment need thresholds were lowered to 5 and 4, the kappa scores also reduced but retained moderate reliability.

### Treatment need according to the GBON and AC indices

It is argued that an aesthetic index used to assess the appearance of borderline cases should only include images of borderline cases [8]. By its nature, it is evident few photos in the AC would qualify as borderline DHC 3. It has also been suggested that the AC treatment threshold be lowered [8–10]. In light of the controversy regarding AC thresholds for access to publicly funded orthodontic treatment, it was appropriate to compare the GBON with the AC at different thresholds of treatment need (AC 6 as currently used in primary care in England and Wales, AC 5 [8], and AC 4 [9, 10]). As the AC treatment need threshold was lowered, the number of malocclusions falling into the treatment need category increased, as did the levels of agreement with the GBON.

When using the GBON, the assessors found 14 out of the 30 malocclusions as needing treatment (by selecting photos B, D, F, G or H) and 16 as not needing treatment (by selecting photos A, C or E). At AC treatment need threshold 4, the malocclusions considered to need treatment were similar to those identified using the GBON. At treatment threshold 6, the AC identified just 3 malocclusions out of 30 as needing treatment, thereby failing to identify most malocclusions considered to need treatment. This supports previously conducted research advocating a reduction in the AC treatment need threshold [8–10].

### Representation of borderline malocclusion traits

The six features of a borderline malocclusion (IOTN DHC 3) are clearly stated, yet three of these features are not represented by photos in the AC (reverse overjets, cross bites and open bites). Crossbites are also not represented by the GBON except as part of a reverse overjet. All these traits, however, were considered to need treatment.

### Ease of use and appropriateness of the GBON index

Male and female orthodontists found the GBON both easier to use and more appropriate for judging borderline malocclusions, despite never having used it before. This indicates that the GBON Index is easy to grasp and could be applied in everyday practice.

Of the subgroup that had previously attended an IOTN calibration course, fewer considered the GBON easier to use (46.6%) compared to the 66.7% of the non-calibrated assessors who found it easier. On the other hand, 69.8% of the calibrated and 72.9% of the non-calibrated assessors felt the GBON was more appropriate in judging borderline malocclusions, despite not being made aware that the malocclusions being assessed were all borderline DHC 3.

### Limitations

A weakness shared by GBON and AC is that overjets are difficult to assess aesthetically from an anterior photograph. Ideally, a clinical assessment of the patient is required where other important aspects of dental aesthetics can be taken into consideration.

Repeating 10 of the photographs at the end of the series was considered an appropriate way to carry out the error study. There is a possibility that some malocclusions may have been recalled by assessors taking part in the study, though perhaps not the scores given.

## Conclusions

Both GBON and AC had good and similar inter- and intra-examiner reliability.

The GBON identified a greater number of DHC 3 malocclusions as needing treatment than the AC except when the treatment need threshold is lowered to 4. At this level, there was substantial agreement between the indices.

The GBON identified reverse overjets and anterior open bites as needing treatment but the AC, at threshold 6, did not identify anterior open bites as needing treatment.

Despite a lack of familiarity, the new GBON index was considered easier to use and more appropriate for judging borderline cases than the AC index.

## Data Availability

The datasets generated and analysed during the current study are not publicly available due them being collected on paper but may be available from the corresponding author on reasonable request.
